# Self-Adaptive MOEA Feature Selection for Classification of Bankruptcy Prediction Data

**DOI:** 10.1155/2014/314728

**Published:** 2014-02-23

**Authors:** A. Gaspar-Cunha, G. Recio, L. Costa, C. Estébanez

**Affiliations:** ^1^Institute of Polymers and Composites-I3N, University of Minho, Guimarães, Portugal; ^2^Department of Computer Science, Universidad Carlos III de Madrid, Leganes, Madrid, Spain; ^3^Department of Production and Systems Engineering, University of Minho, Braga, Portugal

## Abstract

Bankruptcy prediction is a vast area of finance and accounting whose importance lies in the relevance for creditors and investors in evaluating the likelihood of getting into bankrupt. As companies become complex, they develop sophisticated schemes to hide their real situation. In turn, making an estimation of the credit risks associated with counterparts or predicting bankruptcy becomes harder. Evolutionary algorithms have shown to be an excellent tool to deal with complex problems in finances and economics where a large number of irrelevant features are involved. This paper provides a methodology for feature selection in classification of bankruptcy data sets using an evolutionary multiobjective approach that simultaneously minimise the number of features and maximise the classifier quality measure (e.g., accuracy). The proposed methodology makes use of self-adaptation by applying the feature selection algorithm while simultaneously optimising the parameters of the classifier used. The methodology was applied to four different sets of data. The obtained results showed the utility of using the self-adaptation of the classifier.

## 1. Introduction

Bankruptcy prediction has become an important economic phenomenon [1, 2]. The high individual, economical, and social costs arising from bankruptcies have motivated further effort in understanding the problem and finding better prediction methods. In finances, bankruptcy prediction is an important topic of research as it provides a way of identifying business failure, that is, situations in which a firm or particular cannot pay lenders, preferred stock shareholders, suppliers, and so forth. An organisation which is unable to meet its scheduled payments when estimations of future cash show that the current financial situation will not change in the near future is said to undergo into financial distress. Signs of financial distress are evident long before bankruptcy occurs. Research in bankruptcy prediction started in [3] where a univariate discriminant model was used. This was followed by studies using traditional statistical methods which include correlation, regression, logistic models, and factor analysis [4, 5]. More recently, an overview of the classic statistical divided them into four types: univariate analysis, risk index models, multivariate discriminant analysis, and conditional probability models [6].

Modern bankruptcy prediction models combine both statistical analysis and artificial intelligence techniques improving then the decision support tools and decision making [7–9]. In this manner, back propagation artificial neural networks have been applied to bankruptcy prediction [10] whose results revealed better accuracy than predictions made using some other techniques (recursive portioning, *k*-nearest neighbours, C4.5, etc.). Consequently, research has focused on the combination of artificial neural networks with other soft computing tools such as fuzzy sets, genetic programming, ant colony optimisation, or particle swarm optimisation [11–14].

Support vector machines (SVMs) have been largely used for classification and pattern recognition applications. SVMs are a family of generalised linear classifiers widely used for classification of financial data. In particular, several studies have been published on the application of SVMs to the problem of bankruptcy prediction [15–18]. A survey on support vector machines applied to the problem of bankruptcy prediction can be found in [19]. It is worth mentioning that support vector machines require solving a quadratic programming problem which is time consuming when considering large dimensional problems and also that it requires the optimisation of algorithm parameters which may affect its performance. The aim behind this research is to overcome the above limitations which will be accomplished by using feature selection and self-adaptation of the classification algorithm parameters.

Feature selection can be described as one of the initial stages of a classification process by which the complexity of the problem is reduced by elimination of irrelevant features [20]. Feature selection must be approached with the minor lose of information of the original set after the noisy or irrelevant features are removed; that is, the elimination of irrelevant features should not reduce the overall classification accuracy. Being *X* the original set of *n* features for a given classification task, the continuous feature selection problem consists in assigning weights *w*_*i*_ to each feature *x*_*i*_ ∈ *X* in such a way that the order corresponding to its theoretical relevance is preserved. In a similar way, the binary feature selection problem refers to the assignment of binary weights that leads to a reduced subset *X*′⊆*X* of *m* features (with *m* < *n*). In the general case, all features take part in the learning process, each one with a particular contribution. In binary feature selection, only a subset of the features is considered in the learning process for which all of them contribute in the same manner. For the purpose of this work, binary feature selection will be used. In [21], the problem of binary feature selection was formally defined, which, for the general case, consists in finding a compromise between minimising the number of features in *X*′ and maximising an evaluation measure over the subset *J*(*X*′). Notice that an optimal subset of features is not necessarily unique which has motivated further research into this field. Also, there are many potential benefits of feature selection [22], that is, facilitating data visualisation and understanding, reducing the measurement and storage requirements, reducing training and using times, and so forth. Traditional feature selection methods used in bankruptcy prediction consist on applying statistical methods, such as *t*-test, correlation matrix, stepwise regression, principal component analysis, or factor analysis to examine their prediction performance [23]. The application of artificial intelligence techniques, such as evolutionary computation, to the problem of feature selection is now emerging in order to enhance the effectiveness of traditional methods [20].

The general case for feature selection fits into a multi-objective optimisation approach where the aim is to simultaneously optimise two or more conflicting objectives. In addition, identifying a set of solutions representing the best possible trade-offs among objectives of the problem instead of a single solution might be of interest in many cases. Within this context, evolutionary algorithms constitute a preferred choice as they simultaneously deal with a set of solutions, referred to as population, which allows several different solutions to be generated in a single run. Several evolutionary multi-objective approaches (MOEAs) have been applied to finances and economics. The most popular application of MOEAs in the literature deals with the portfolio optimisation problem [24–26], although MOEAs have also been successfully applied to stock ranking [27], risk-return analysis [28, 29], and economic modelling [30, 31]. In a sense, this work constitutes a study on the consequences of simultaneously optimised two or three objective functions over real-world benchmark problems.

Another issue that will be considered in this work is the self-adaptation of the classifier algorithm parameters. Self-adaptation aims at finding suitable adjustment of the algorithm parameters efficiently [32]. In general, the definition of self-adaptation in evolutionary algorithms refers to the adjustment of control parameters that are related to evolutionary routines [33], that is, mutation or crossover rates, population size, and selection strategy. In this work, the scope of this definition will be modified and the aim will be the automatic adjustment of the classification process parameters, which in the present case include the training method, the training fraction, and the specific SVM parameters (e.g., kernel and regularisation parameters). Some other recent works that might be of interest for the reader are [34–38].

The aim of this work is to further investigate into the feature selection problem in bankruptcy prediction using a multi-objective approach, including self-adaptation of the classification algorithm parameters. This work is expected to contribute by introducing a novel multi-objective methodology for feature selection which provides a solution to the problem of bankruptcy prediction compromising both the minimisation of the number of features selected and the maximisation/minimisation of a quality measure of the classifier, for example, accuracy or error. Also, this paper will help to create a better understanding of the application of SVMs to real-world data. The proposed methodology will be validated using bankruptcy prediction datasets found in the literature.

The remaining of this paper is organised as follows. The proposed methodology will be described in detail in Section 2, for which the corresponding expertise areas of classification, using SVMs, feature selection in classification and multi-objective evolutionary optimisation will be introduced.  Section 3 describes the datasets used during the experimental part of this research. Discussion on the performance of the algorithm will follow in Section 4. The paper finalises in Section 5 by pointing out the main contributions, limitations and further extensions to this work.

## 2. Multiobjective Feature Selection

### 2.1. Feature Selection

As stated above, the feature selection problem consists in finding the minimum number of features that are necessary to evaluate correctly a set of data. Considering *X* as the original set of features with a cardinality |*X*| = *n*, the following definition applies [39].

**Definition 1 (feature selection)**

Let *J*(*X*′) be an evaluation measure to be optimised (considering here a maximisation problem, without loss of generality) defined as *J* : *X*′⊆*X* → ℝ. The selection of a feature subset can be seen under three considerations.

- Set |*X*′| = *m* < *n*. Find *X*′ ⊂ *X*, such that *J*(*X*′) is maximum.
- Set a value *J*_0_, that is, the minimum *J* that is going to be tolerated. Find the *X*′⊆*X* with smaller |*X*′|, such that *J*(*X*′) ≥ *J*_0_.
- Find a compromise among minimising |*X*′| and maximising *J*(*X*′) (general case).

In the present work, a wrapper approach was used [40]. Usually, the existing data is divided in two sets, the training and the test data. For that purpose, the existence of (i) a representative set of data, capable of allowing the identification of the relations between the features and the classification of such data, (ii) an algorithm able to classify the data accurately (classification algorithm), (iii) and an optimisation algorithm able to find the best set (or the minimum) of features that classify the data with the best accuracy and/or the minimum error is necessary.

**Figure 1:**
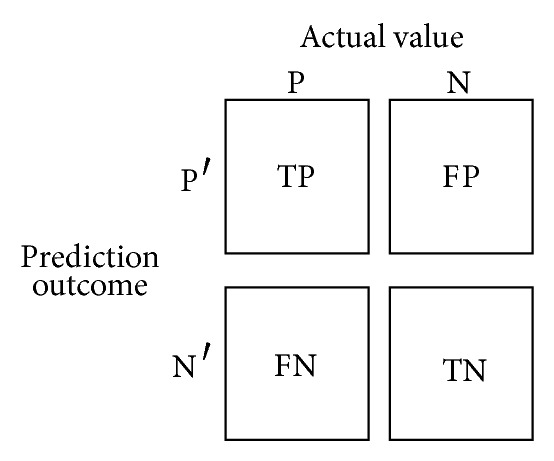
Confusion matrix.

**Figure 2:**
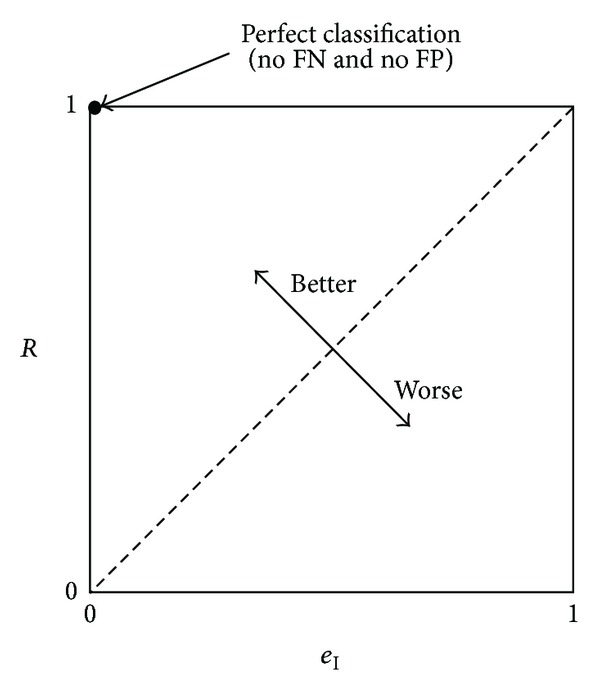
ROC curve.

Figure 1 illustrates the well-known confusion matrix, for a situation with two classes. TP (true positives) are the positive instances correctly classified, TN (true negatives) are the negative instances correctly classified, FP (false positives) are the positive instances incorrectly classified, and FN (false negatives) are the negative instances incorrectly classified. Based on this taxonomy, different measures can be defined to quantify the accuracy and the error achieved by the classifier as follows:

(1) $$\begin{array}{c} \text{Acc}=\frac{\text{TP}+\text{TN}}{\text{TP}+\text{FP}+\text{TN}+\text{FN}}\\ R=\frac{\text{TP}}{\text{TP}+\text{FN}}\\ P=\frac{\text{TP}}{\text{TP}+\text{FP}}\\ e_{\text{I}}=\frac{\text{FP}}{\text{FP}+\text{TN}}\\ e_{\text{II}}=\frac{\text{FN}}{\text{TP}+\text{FN}}\\ F_{m}=\frac{2\cdot P\cdot R}{P+R}, \end{array}$$

where Acc is the accuracy, *R* is the recall or sensitivity, *P* is the precision, *e*_I_ and *e*_II_ are the classification errors of types I and II, respectively, and *F*_*m*_ is the harmonic mean of the sensitivity (*R*) and precision (*P*). After the above formalism, the problem consists in maximising Acc, *R*, *P*, and *F*_*m*_ and minimising the errors. There are other type of classification measures that can be also applied. However, the problem to be addressed is the simultaneous optimisation of some of these measures. For example, in bankruptcy prediction, the maximisation of the profits, but, simultaneously, the minimisation of losses is desired. In the present situation the profits can be quantified by recall (*R*), since it is a direct measure of the positives correctly classified (TP), that is, the companies that test *well* and are *healthy*, and the losses can be quantified by the error of type I (*e*_I_), a measure of the positives incorrectly classified (FP), that is, the companies that test *well* but actually are in *bankruptcy*. The trade-off between *R* and *e*_I_ is known as the Receiver Operating Characteristics (ROC) curve [41, 42].  Figure 2 illustrates this concept. The ideal point is identified by “1” and means a perfect classification.

The above example illustrates the importance of optimising more than one objective simultaneously. In fact, in the case of feature selection, the first objective to be optimised (minimised) is the number of features that are necessary to get an accurate classification, which can be taken into account by maximising Acc or *F*_*m*_, for example, but also by obtaining the best trade-off between *R* and *e*_I_, as illustrated in Figure 2. In the first case, there are two objectives to be satisfied simultaneously, while, in the second, three objectives are considered. Therefore, the use of a multi-objective optimisation algorithm together with an accurate classifier is of primordial importance.

### 2.2. Support Vector Machines

There are available in the literature a large number of algorithms/methods for classification of data. For example, the WEKA software offers a great number of different methods ready to be used in an straightforward way [43]. A good survey about the best classification algorithms can be found in [44].

The method adopted here is the Support vector machines (SVMs). SVMs are a set of supervised learning methods based on the use of a kernel, which can be applied to classification and regression [45]. In the SVM, a hyperplane or set of hyperplanes are constructed in a high-dimensional space. The initial step consists in transforming the data points, through the use of nonlinear mapping, into the high-dimensional space. In this case, a good separation is achieved by the hyperplane that has the largest distance to the nearest training data points of any class. Thus, the larger this margin, the smaller the generalisation error of the classifier. SVMs can be seen as an extension to nonlinear models of the generalised portrait algorithm developed in [46]. For the purpose of this work, the SVM package from LIBSVM was used [47].

The SVMs depend on the definition of some important parameters. First, it is necessary to select the the type of kernel. In the present work, the Radial Basis Function (RBF) kernel was adopted due to its efficiency. Then, it becomes necessary to select the SVM type which depends on its usage, that is, if it is used for classification or regression. Since this work deals with classification, the *μ*-SVC and *C*-SVC methods were selected. Both, the kernel and the type of SVM, depend on the value defined for some parameters that must be carefully set, the kernel parameter (*γ*) and the regularisation parameter that depends on the type of SVM chosen (*μ* and *C*). Finally, some other parameters were studied including the training method and the training fraction. Two different training methods were tested, the holdout method, where a fraction (training fraction) of the instances are used to train the SVM and the remaining are used for testing and the *k*-fold method, that consists in dividing the set of instances in *k* subsets. Then *k* − 1 subsets are used to train the SVM and the remaining set is used for validation. The process is repeated *k* times, accounting for all subsets used for validation, and the accuracy is obtained as the average of the *k* training/testing steps [47].

Due to the large number of parameters that must be set before applying the optimisation algorithm, it makes sense to apply the feature selection algorithm and the optimisation of these parameters simultaneously. This is what is done in the present work. Therefore, the following parameters were optimised simultaneously with the process of feature selection: training method (holdout, H; or 10-fold, *K*(10), validation), training fraction (TF), kernel (*γ*), and regularisation parameters (*μ* or *C*). More details about the implementation of this strategy are given in the next subsection.

### 2.3. Multiobjective Evolutionary Algorithm

In order to deal with multiple objectives multiobjective optimisation algorithms (MOOA) must accomplish two basic functions simultaneously: (i) they need to guide the population towards the optimal Pareto set. This can be done by using a fitness assignment operator that takes into account the non-dominance concept. (ii) The nondominated set must be maintained as diverse as possible; that is, the solutions must be well distributed along the entire optimal Pareto front. Additionally, it is also necessary to maintain an archive of the best solutions found during the various generations in order to prevent some nondominated solutions from being lost. Therefore, generally in MOEAs, it is only necessary to replace the selection phase of a traditional EA by a routine able to deal with multiple objectives [48, 49].

In this work, the MOEA adopted is the reduced Pareto set genetic algorithm (RPSGA) [50]. However, any other multi-objective algorithm can be used for the same purpose. The main steps of this algorithm are described below (Algorithm 1). The algorithm starts by the random creation of an internal population of size *N* and an empty external population of size 2*N*. Then, at each generation (i.e., while the stopping criteria are not met), the following operations are performed: (i) the internal population is evaluated using the SVM routine; (ii) a clustering technique is applied to reduce the number of solutions on the efficient frontier and to calculate the ranking of the individuals of the internal population; (iii) the fitness of the individuals is calculated using a ranking function; (iv) a fixed number of the best individuals is copied to the external population; (v) if the external population is not totally full, the genetic operators of selection, crossover, and mutation are applied to the internal population to generate a new population; (vi) when the external population becomes full, the clustering technique is applied to sort the individuals of the external population, and a predefined number of the best individuals is incorporated in the internal population by replacing lowest fitness individuals.

Detailed information about this algorithm can be found in [50, 51]. The influence of some important parameters of the algorithm, such as size of internal and external populations, number of individuals copied to the external population in each generation and from the external to the internal population, and the limits of the indifference of the clustering technique, had already been studied and the best values have been suggested [50].

### 2.4. Methodology for Feature Selection

The linkage between the problem to solve (the selection of features), the SVM, and the MOEA is done as follows. During the generation of the initial population, the chromosome (generated randomly) is constituted by a binary string identifying if the corresponding feature is present (value equal to 1) or not (value equal to 0) and the values of the classification algorithm/process (TF, H or *K*(10), *γ* and *μ*, or *C*), which are used for self-adaptation. These chromosome values are then passed to the SVM during the evaluation of the population. The SVM returns the achieved values of accuracy and errors (1) obtained with the selected features and parameter values that are present in the chromosome of each individual.

The RPSGA algorithm was adapted to deal with the above feature selection problem. With respect to the classifier parameters, two approaches were considered. Initially, a pure feature selection problem was analysed where these parameters were not allowed to vary after being set up at the beginning of the algorithm. In a second approach, these parameters were included in the chromosome as variables to be optimised. The latter approach has the advantage of obtaining in a single run the best features and, simultaneously, fine tuning the classifier parameters (self-adaptation). Each candidate solution generated by the RPSGA will be externally evaluated by the SVM whose result will be returned to the RPSGA to be used as fitness in the genetic routine. New solutions will be generated based on the performance of the previous generation. As usual, the fittest solutions have more possibilities of survival.

## 3. Datasets

In the present study, the four datasets presented below will be used to validate the proposed methodology. Note that the DIANE data consists of two datasets from different years.

### 3.1. Industrial French Companies Data

In the present work, two samples, from the years 2005 and 2006, respectively, obtained from the DIANE database were selected. The original database comprised financial ratios of about 60 000 industrial French companies with at least 10 employees. The dataset includes information about 30 financial ratios of companies covering a wide range of industrial sectors (see Table 1). Since the original database contained many instances with missing values, especially, concerning defaults companies, the default cases were sorted by the number of missing values and only samples with less than 10 missing values were selected. A final set of 600 default examples was obtained. In order to create a balanced dataset, 600 random nondefault examples were selected and added to the dataset, thus resulting in a set of 1200 examples. Similar preprocessing of this dataset can be found in [31, 52, 53].

### 3.2. German Credit Data

The German Credit database was created at the University of Hamburg and is publicly accessible at the UCI Machine Learning Repository [54]. It consists of 1000 instances of credit applications which are described by the 20 attributes shown in Table 2. Examples of previous usage of the German Credit dataset can be found in [55, 56]. There are two versions of the German dataset available, the original German Credit dataset which consists of numerical and nominal attributes and its numeric version produced at the Strathclyde University. As the method proposed in this paper only accepts numerical attributes, the numeric version of the data will be used.

### 3.3. Australian Credit Data

The Australian Credit database originates from [57] and concerns data form 690 credit card applications. The data are publicly available in the UCI Machine Learning Repository [54]. Each instance consists of 14 attributes and one of two possible classes (all attribute names and values were changed to meaningless symbols to protect the confidentiality of the data). The class distribution is similar for both, 44.5% versus 55.5%. Examples of previous usage of this dataset can be found in [58].

### 3.4. Data Normalisation

In general, a large amount of data is available and often these data are inconsistent and redundant being necessary considerable manipulation to make it useful for problems like credit risk analysis. It becomes important to identify the ratios or ranges of data that are relevant to the problem. Restricting the data to the relevant ranges represents an advantage to reduce the complexity of the problem.

Due to the large diversity of data concerning the type of data (e.g., real or integer values, numeric or categorical) and the range of variation of the values for each feature, some normalisation of the data becomes necessary. Therefore, the data was transformed as follows:

(1) logarithmic transformation:
  (2) $$\begin{array}{c} x_{ij}^{\prime }=\{\begin{array}{cc} \log\left(x_{ij}+1\right) & x_{ij}\geq 0\\ -\log\left(-x_{ij}+1\right) & x_{ij}<0, \end{array} \end{array}$$
(2) centering and standardizing the data:
  (3) $$\begin{array}{c} x_{ij}^{\prime \prime }=\frac{x_{ij}^{\prime }-\overset{-}{\text{A}}\text{VG}\left(x_{j}^{\prime }\right)}{\text{STD}\left(x_{j}^{\prime }\right)}, \end{array}$$
(3) normalisation of the data in the interval [−1, 1]:
  (4) $$\begin{array}{c} y_{ij}=2\frac{x_{ij}^{\prime \prime }-\mathrm{Min}\left(x_{ij}^{\prime \prime }\right)}{\mathrm{Max}\left(x_{ij}^{\prime \prime }\right)-\mathrm{Min}\left(x_{ij}^{\prime \prime }\right)}-1, \end{array}$$

where *i* represents the instance, *j* stands for feature, *x*_*ij*_ is the original data in a matrix form (which is transformed successively in *x*_*ij*_′′ and *x*_*ij*_′′), AVG (*x*_*j*_′) and STD (*x*_*j*_′) are the average and the standard deviation of all instances for feature *j*, respectively, and *y*_*j*_ is the final value used by the classifier. The data used by the classifier is restricted to the interval [−1, 1] as recommended in [44].

## 4. Results and Discussion

### 4.1. Computational Experiments

Table 3 presents the set of experiments carried out to test the proposed approach. Due to the stochastic nature of the evolutionary algorithm, 10 runs (*a*, *b*,…, *j*) for each experiment were performed, using different seed values (as required by the random number generator). In the case of Experiment 1, the C-SVC method was used with the following fixed parameters: holdout (H) validation as training method, TM, training fraction, TF, equal to 0.7, kernel parameter, *γ*, equal to 0.1, and the regularisation parameter, *C*, equal to 10. In the remaining experiments, these parameters were allowed to range in the following intervals: *γ* ∈ [0.005,10], *C* ∈ [1,1000], *ν* ∈ [0.01,0.5], TM ∈ [*H*  or  *K*(10)], and TF ∈ [0.5,0.8]. In Table 3, *N*1 represents the maximum number of features allowed in the initial generation, that is, if *N*1 is equal to 5 means that in the initial generation the individuals of the population have at the most 5 features. In consecutive generations, the number of selected features was allowed to grow until the maximum of features for each database is reached: for French industrial companies, subscript F, *N*_max⁡_ = 20; for German Credit data, subscript G, *N*_max⁡_ = 20; and for Australian Credit data, subscript A, *N*_max⁡_ = 14. Besides, Figures 1 and 2 should not become a problem (with respect to the dataset dimension) for standard SVMs experimentation; this work tries to demonstrate that feature selection is useful for the application of SVMs over datasets of high dimension.

The aim of Experiment 1 is to compare the performance of the feature selection method proposed when the classifier parameters are fixed to that of the same method when the parameters are allowed to vary. This will be done by comparing Experiments 1, 2, and 12. Experiments 2 to 7 are thought to illustrate the influence of the method when different classification measures are applied. In the case of Experiments 8 to 11, the aim is to study the influence of the maximum number of features of the initial population (*N*1) in the evolution of ROC curves (i.e., *R* versus *e*_I_). Finally, Experiments 12, 13, and 14 were intended to show the influence of the SVM method used. In all runs, the following RPSGA parameters were used (see [50] for more details): the main and elitist population sizes were 100 and 200 individuals, respectively; fitness proportional selection was adopted; crossover rate of 0.8 and mutation probability of 0.05 were used; the number of ranks was set to 30 and the limit of indifference of the clustering technique was set to 0.01, whereas the number of generations was set to 100 for all runs.

### 4.2. Analysis of a Standard Experiment

**Figure 3:**
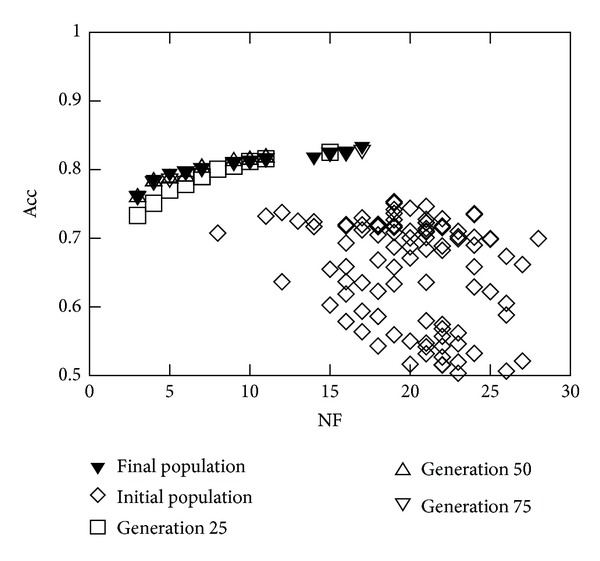
*Run-a* of Experiment 2 (initial population and nondominated solutions of the final population).

This section is aimed at showing the type of results that can be obtained using the proposed methodology. For that purpose, Figure 3 shows the entire initial population and the nondominated solutions corresponding to generations 25, 50, 75, and 100 for *Run-a* of Experiment 2. This graph presents the trade-off between Acc (to be maximised) and NF (to be minimised). It can be easily observed that the algorithm is able to evolve the population significantly, from the initial population (randomly generated), located predominantly at the bottom right corner, towards the top left corner. It is also noticeable that only 50 generations are needed to reach a reasonable approximation of the Pareto front. The use of 100 generations was only used to guarantee the convergence of the algorithm.

Table 4 shows the obtained results corresponding to the decision variable domain for the above run after 100 generations. The accuracy is ranged between 76.3% and 83.5%, when considering a minimum number of 3 features and a maximum of 17, respectively. In all cases, the holdout (H) cross validation training method was selected and the training fraction lies around 52% and *γ* is ranged between 0.13 and 0.55, whereas *C* fluctuate between 10 and 211. This indicates that decision variables (TM, TF, *γ*, and *C*) converge for a small interval when compared to the initial range where they are allowed to vary.

**Figure 4:**
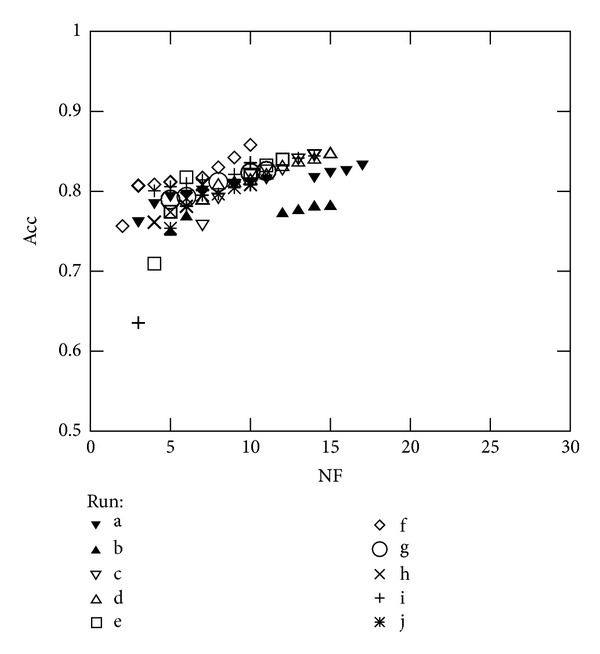
All runs of Experiment 2 (nondominated solutions of the final population).

However, the target consists in finding better solutions than those obtained over a single run.  Figure 4 shows the optimal Pareto curves of the 10 runs that were performed for Experiment 2. It can be seen that there is one of these runs that dominates the others, *Run-f*, except when NF = 6, where the best solution is obtained for *Run-e*.  Table 5 shows the decision variable values of the corresponding Pareto front, for which Acc is ranged between 75.6% and 85.8%, the obtained TM is hold out for all cases, and the TF lies around 75%. On the other hand, the SVM parameters have a large variation which indicates that *γ* and *C* play an important role in acquiring best accuracies. Similar conclusions can be drawn when analysing the results obtained using the remaining datasets.

### 4.3. Analysis and Comparison of Results

**Figure 5:**
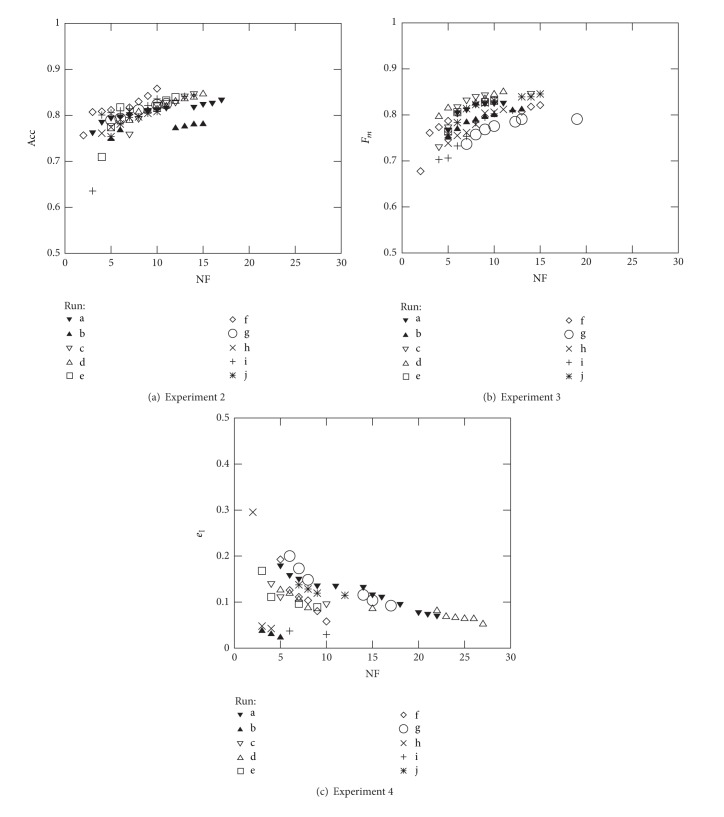
Optimal Pareto fronts for Diane 2005 data (10 runs).

**Figure 6:**
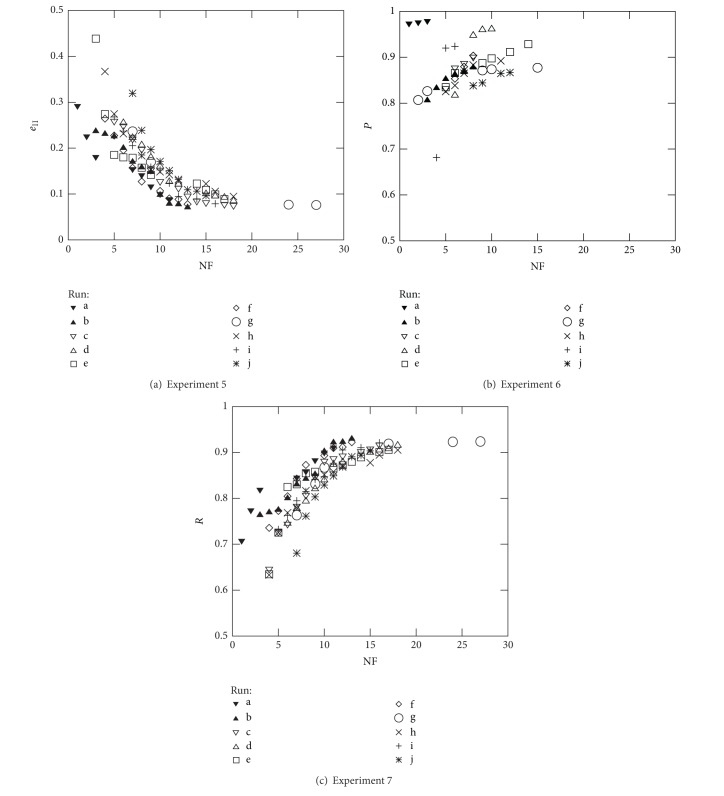
Optimal Pareto fronts for Diane 2005 data (10 runs).

Figures 5 and 6 represent the nondominated solutions of the 10 different runs carried out in Experiments 2 to 7 using to the French industrial companies in 2005 dataset. These plots allow to assess the efficiency of the proposed optimisation methodology when dealing with all the objective function measures presented in Section 2. As expected, and since the common objective used in these experiments is the minimisation of NF, the solutions evolve nicely towards the region where the true Pareto front is supposed to be; that is, when simultaneously maximising a second objective (e.g., Acc, *F*_*m*_, *R*, and *P*) the solutions evolve towards the top left corner, while when simultaneously minimising a second objective (e.g., *e*_I_ and *e*_II_), the solutions evolve towards the bottom left corner.

Further analysis of Figures 5 and 6 helps to identify the ranges that can be accomplished when using the different objective functions (for the French datasets): Acc and *F*_*m*_ ∈ [70%, 85%], *P* ∈ [80%, 100%], *R* ∈ [60%, 95%], *e*_I_ ∈ [0%, 20%], and *e*_II_ ∈ [5%, 35%]. However, when considering the best values in a particular run, the following values were found: Acc = 85.8%, *F*_*m*_ = 85.0%, *e*_I_ = 2.3%, *e*_II_ = 7.1%, *P* = 97.9%, and *R* = 92.9%, corresponding to NF equal to 10, 11, 5, 13, 3, and 13, respectively. Considering a given number of features, for example, NF = 10, the following best values are found: Acc = 85.8%, *F*_*m*_ = 84.4%, *e*_I_ = 3.0%, *e*_II_ = 9.8%, *P* = 96.1%, and *R* = 90.2%. On the other hand, when considering all ten runs of each experiment, the variation range for each objective function can be graphically observed. Such a variation enforces the use of several runs with different seed values in order to select the best set of features as well as the best classifier parameters. Since the final accuracy will depend certainly on the combination of the right features, the methodology adopted cannot be based on selecting the features that appear more frequently in the 10 runs performed for each experiment [59].

**Figure 7:**
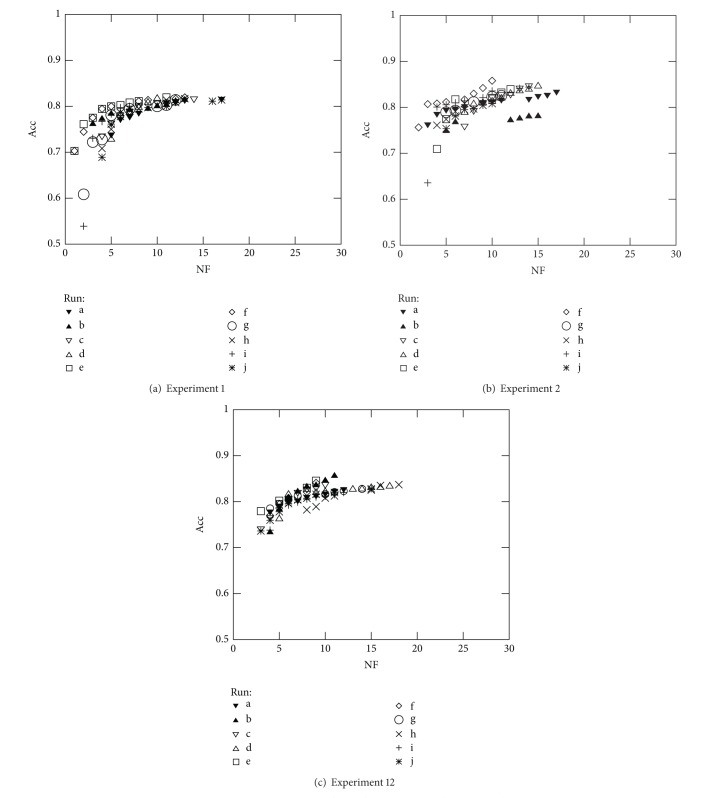
Optimal Pareto fronts for Diane 2005 data (10 runs).

**Figure 8:**
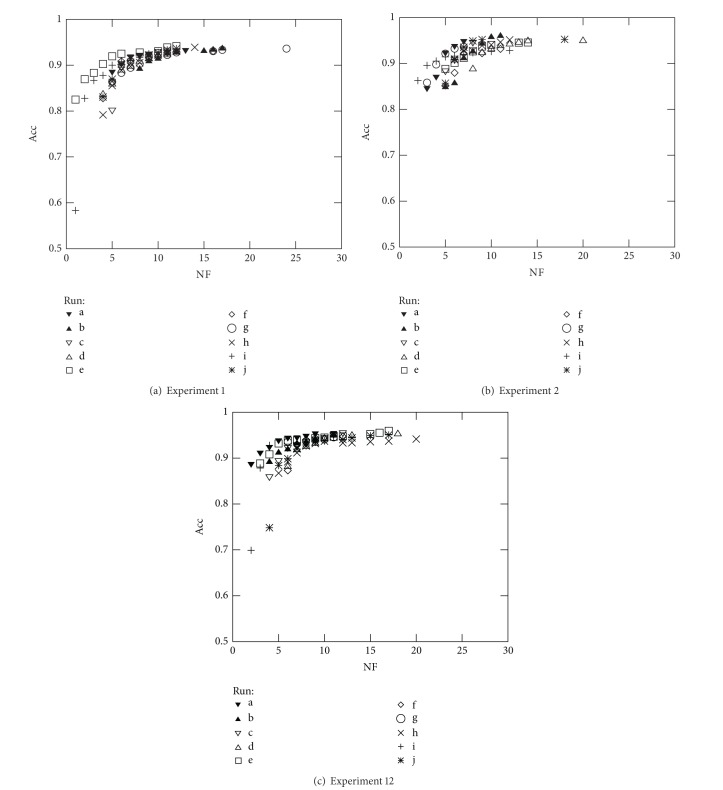
Optimal Pareto fronts for Diane 2006 data (10 runs).

**Figure 9:**
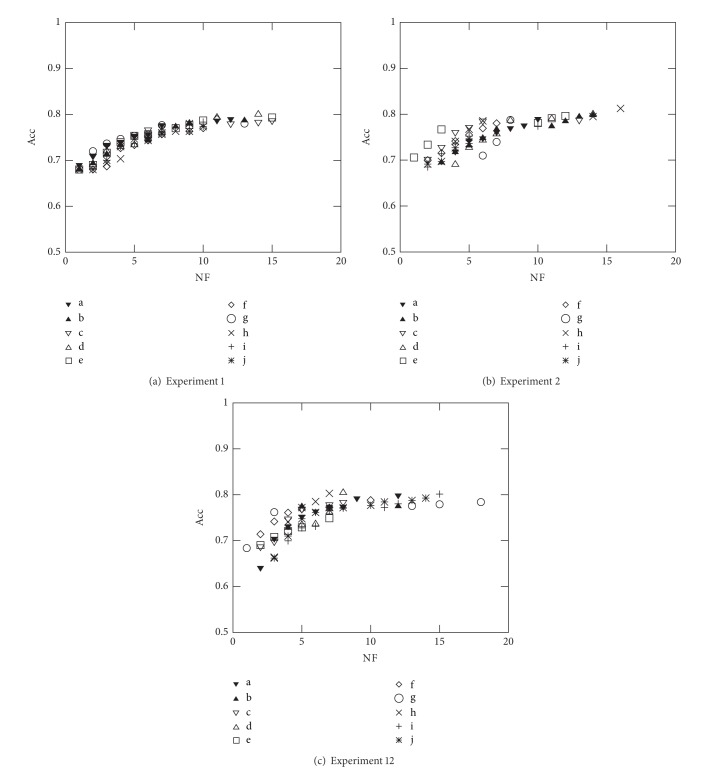
Optimal Pareto fronts for German Credit data (10 runs).

**Figure 10:**
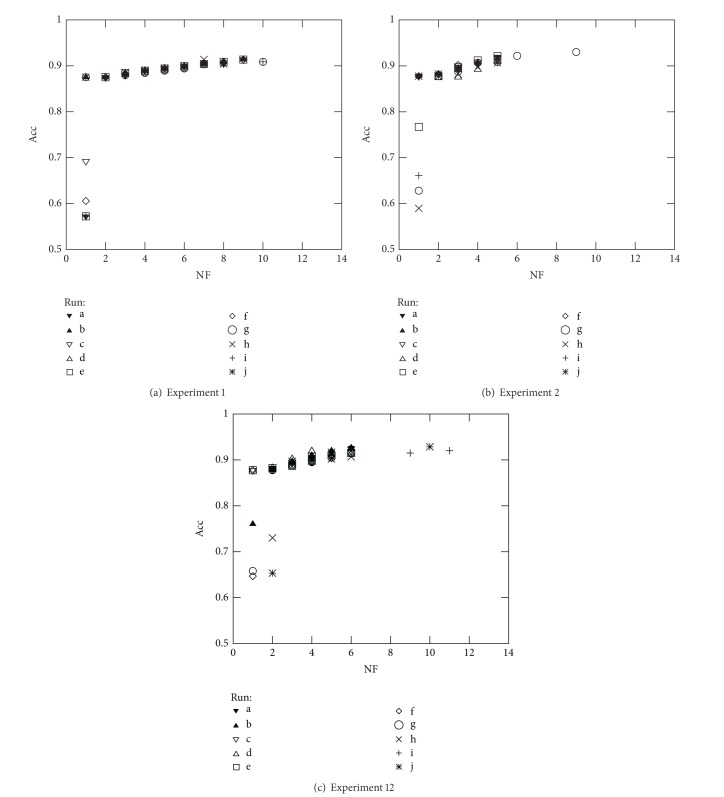
Optimal Pareto fronts for Australian Credit data (10 runs).

The above reasoning was used to select the best solution of the front when comparing the results from Experiments 1, 2, and 12 over all datasets studied. Note that Experiments 1, 2, and 12 consist on simultaneously optimising NF and Acc (see Figures 7, 8, 9, and 10). Furthermore, the above analysis allowed to create Table 6 which summarises the solutions found for three different cases: solutions with best accuracy (Best) and best solutions using only 5 (NF ≤ 5) and 10 (NF ≤ 10) features, respectively.

As expected and in general, the results of Table 6 show that the best accuracy is obtained when the classifier parameters are also optimised (Experiments 2 and 12). Concerning the use of the C-SVC or the *μ*-SVC kernels, no definitive conclusion can be drawn, since the C-SVC kernel yielded the best result for Diane05, whereas the *μ*-SVC kernel yielded the best result the the Australian data and for some other cases the best result depends on the number of features (Diane06 and German data). With respect to the runs where the “best” results were obtained for each of the three conditions that were analysed, again there is some variability; in some cases, the results were taken from the same run but in most of the cases they were not. Again, this fact was expected after the analysis made in the previous section. In all cases, the holdout validation method is selected, TF ranges between 70% and 80% in most cases (except in the case of the German database), and the kernel and regularisation parameters have a high variability to maximise the accuracy. This was also expected after the analysis of the previous section.

The analysis or results show that the desired accuracy can be achieved using several combinations of features. Results coming from the same run tend to select the same features (this fact was also observed in the results presented in Tables 4 and 5). An interesting finding came from Experiment 2 over Diane05 database; it was observed that when the number of features was reduced to 5 at the most (NF ≤ 5), four out of five of the features selected were identical to one of the features that were selected for the best solution condition (features 7, 13, 21, and 23), but the last feature selected when using this constraint was not included in the best solution (feature 29). Many valuable information can be obtained from Table 6. As an example, if the problem consists on obtaining the best accuracy using five features at the most (NF ≤ 5), the features identified in bold should be selected to be used in future classifications together with their corresponding parameter for each dataset considered.

**Figure 11:**
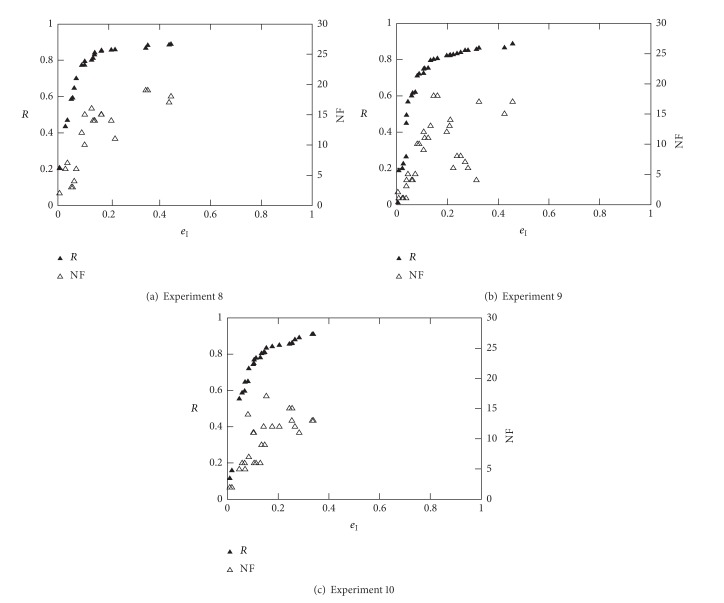
ROC curves for Diane 05 data (best of 10 runs).

**Figure 12:**
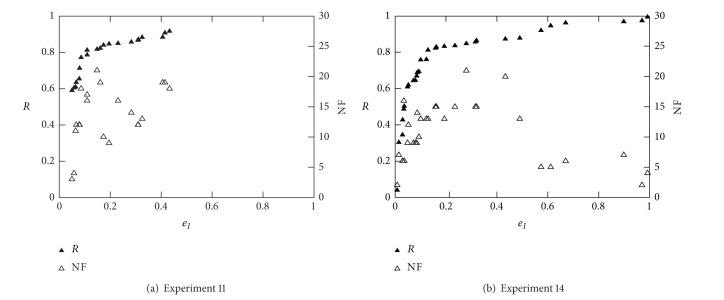
ROC curves for Diane 05 data (best of 10 runs).

Figures 11 and 12 show the best results achieved in Experiments 8, 9, 10, 11, and 14. Note that these experiments consist in optimising three different objectives (*R*, *e*_I_, and NF) and were aimed to obtain the results that best fit in a ROC curve; that is, *R* = *f*(*e*_I_). Besides the optimisation that was carried out considering all three objectives, only nondominated solution with respect to objectives *R* and *e*_I_ are presented (best of 10 runs for each experiment).  Table 7 shows a summary of results from the above experiments for all databases using two different conditions (*e*_I_ ≤ 10% and NF ≤ 5). The area under ROC was computed at first for all cases and then best results were presented for each condition. Identical conclusions, to that of the beginning of this section, Section 4.3, can be made here concerning the algorithm parameters, that is, best kernel (which depend on the database), best validation method, training fraction, and kernel and regularisation parameters. Similarly, there exist various combinations of features that allow the obtention of the best *R* and *e*_I_ values. As before, the best solutions using five features at the most, NF ≤ 5, can be selected for each database. Such features are identified in bold in Table 7 and can be used in future classification together with their corresponding classifier parameters.

In [60], clustering feature selection methods were used to identify the most relevant features on several datasets. The Australian Credit dataset was used to test three versions of a clustering based algorithm with different optimisation strategies. The structure of clusters, found by the optimisation version of the algorithm proposed in the above paper, indicates the subset of three relevant features for classification, features 8, 9, and 14. Almost all solutions obtained in the experiments carried out in this paper (see Tables 6 and 7) using the Australian data include features 8 and 9. Feature 14 is also present in most of the solutions obtained in this work.

Prediction of financial distress of companies using Diane dataset, was previously analysed using several machine learning approaches [61]. Support vector machines achieved the highest accuracy considering five features selected by SVM attribute evaluation method. The five features selected for predicting failures during 2007 using historical data from 2006, 2005, and 2004 were 1, 4, 11, 16, and 28 (which differs from the solution obtained here). However, it should be noted that these results were obtained using historical data and, therefore, they are not comparable to the results obtained in this work.

An approach to solving classification problems by combining feature selection and neural networks was proposed in [62]. A feature selection algorithm based on the notion of entropy from the information theory was applied to the German Credit dataset yielding the selection of the following seven features: 1, 2, 3, 5, 6, 15, and 20. Authors found that the predictive accuracy was marginally larger with the exclusion of the 13 redundant features. Most of the solutions obtained by the approach presented in this work include some of these features (in particular, features 1, 2, 3, and 5). However, it should be noted that the experimental set-up of the two studies is rather different and, therefore, conclusions must be drawn carefully.

## 5. Conclusion

With the current global economic situation where several countries are getting through economic recession, bankruptcy prediction is acquiring importance as a financial topic of research. When the financial data to be analysed becomes large, the need for feature selection arises as a tool used to reduce both computational times and number of computations by getting rid of irrelevant features. Feature selection also gives a method to evaluate the importance of each feature within the studied dataset.

This work aimed at investigating the feature selection problem in bankruptcy prediction using a multi-objective approach which includes self-adaptation of the classification algorithm parameters. For that purpose, a new methodology has been proposed and its performance has been evaluated using real-world benchmark problem datasets for bankruptcy prediction. A large set of experiments using different objective functions, such as accuracies, error, and sensitivity measures, have been performed which provides a better understanding on the application of SVMs to real-world data. The performance of the proposed method was also studied using two- and three-objective approaches.

Results have shown that the method performs well using the benchmark datasets studied. Large accuracies have been obtained using a significantly reduced subset of features. Consequently, the more the considered features, the lager the accuracies. Also, being a multi-objective technique, instead of a single solution, a set of nondominated solutions is provided which may help the decision maker to evaluate the trade-off in making a sacrifice in one of the objective functions towards obtaining a gain in some others. The inability for the classifier to handle nominal features within the data turned out to be the main limitation of the proposed method. This limitation was inherent to the classifier used by the method; it was overcome by converting nominal attributes of the data to numerical.

A possible extension to this work could be made by taking advantage of the multi-objective nature of the set of solutions and analysing in detail the trade-off between them, thus helping decision makers to choose the preferred solution for their needs. The proposed method could also be extended to work with many objectives as real-world situations actually do.

## Figures and Tables

**Algorithm 1:**
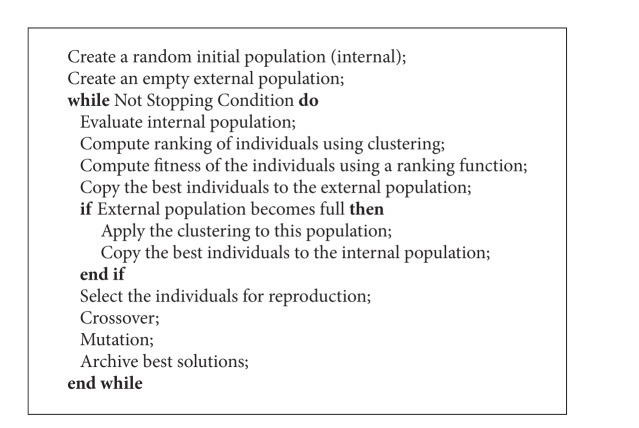
Reduced Pareto set genetic algorithm (RPSGA).

**Table 1:** Set of features considered for the industrial French companies.

Feature	Designation
F1	Number of employees
F2	Capital employed/fixed assets
F3	Financial debt/capital employed
F4	Depreciation of tangible assets
F5	Working capital/current assets
F6	Current ratio
F7	Liquidity ratio
F8	Stock turnover days
F9	Collection period
F10	Credit Period
F11	Turnover per employee (thousands euros)
F12	Interest/turnover
F13	Debt period days
F14	Financial debt/equity
F15	Financial debt/cashflow
F16	Cashflow/turnover
F17	Working capital/turnover (days)
F18	Net current assets/turnover (days)
F19	Working capital needs/turnover
F20	Export
F21	Value added per employee
F22	Total assets/turnover
F23	Operating profit margin
F24	Net profit margin
F25	Added value margin
F26	Part of employees
F27	Return on capital employed
F28	Return on total assets
F29	EBIT margin
F30	EBITDA margin

**Table 2:** Set of features considered for the German Credit.

Feature	Designation
F1	Status of existing checking account
F2	Duration in months
F3	Savings account/bonds
F4	Purpose
F5	Credit amount
F6	Savings account/bonds
F7	Present employment since
F8	Instalment rate in percentage of disposable income
F9	Personal status and sex
F10	Other debtors/guarantors
F11	Present residence since
F12	Property
F13	Age in years
F14	Other instalment plans
F15	Housing
F16	Number of existing credits at this bank
F17	Job
F18	Number of people being liable to provide maintenance for
F19	Telephone
F20	Foreign worker

**Table 3:** Set of computational experiments (*N*1 is the maximum number of features in the initial population; *X* means experiment not done).

Exp	SVM type	Objectives	*N*1 F-G-A
1	C-SVC14	NF + Acc	30-20-14

2	C-SVC01	NF + Acc	30-20-14
3	C-SVC02	NF + *F*_*m*_	30-20-14
4	C-SVC03	NF + *e*_I_	30-20-14
5	C-SVC04	NF + *e*_II_	30-20-14
6	C-SVC05	NF + *P*	30-20-14
7	C-SVC06	NF + *R*	30-20-14

8	C-SVC07	NF + *R* + *e*_I_	30-20-14
9	C-SVC08	NF + *R* + *e*_I_	5-5-5
10	C-SVC09	NF + *R* + *e*_I_	15-15-10
11	C-SVC10	NF + *R* + *e*_I_	25-*X*-*X *

12	*μ*-SVC11	NF + Acc	30-20-14
13	*μ*-SVC12	NF + *F*_*m*_	30-20-14
14	*μ*-SVC13	NF + *R* + *e*_I_	30-20-14

**Table 4:** Optimal solutions for *Run-a* of Experiment 2 (Diane 2005 database).

NF	Features	Acc	TM	TF	*γ*	*C*
3	1, 14, 24	76.3%	H	50.4%	0.501	10.4
4	1, 14, 16, 24	78.6%	H	50.6%	0.403	39.6
5	1, 8, 14, 21, 24	79.5%	H	52.0%	0.280	211.0
6	1, 8, 14, 15, 16, 24	79.8%	H	52.2%	0.297	173.9
7	1, 8, 12, 14, 15, 16, 24	80.3%	H	54.4%	0.543	112.8
9	1, 8, 12, 14, 21, 23, 24, 25, 27	81.2%	H	52.2%	0.134	86.9
10	1, 8, 12, 14, 15, 16, 21, 23, 24, 25	81.4%	H	53.6%	0.343	114.6
11	1, 8, 12, 14, 15, 16, 21, 23, 24, 25, 27	81.7%	H	52.2%	0.164	59.7
14	1, 5, 7, 8, 9, 12, 14, 15, 16, 18, 21, 23, 24, 25	81.9%	H	52.1%	0.539	23.9
15	1, 2, 5, 7, 8, 9, 12, 14, 15, 16, 18, 21, 23, 24, 25	82.5%	H	52.3%	0.384	26.8
16	1, 2, 5, 6, 7, 8, 9, 12, 14, 15, 16, 18, 21, 23, 24, 27	82.8%	H	52.2%	0.405	24.5
17	1, 2, 5, 6, 7, 8, 9, 12, 14, 15, 16, 18, 21, 23, 24, 25, 27	83.5%	H	52.1%	0.354	24.8

**Table 5:** Optimal solutions for Experiment 2 (Diane 2005 database).

NF	Run	Features	Acc	TM	TF	*γ*	*C*
2	f	7, 21	75.6%	H	73.9%	0.066	373.1
3	f	7, 21, 23	80.7%	H	74.5%	0.127	668.5
4	f	7, 21, 22, 29	80.8%	H	74.3%	0.0102	855.8
5	f	7, 13, 21, 23, 29	81.2%	H	74.4%	0.0104	844.4
6	e	6, 12, 13, 19, 21, F29	81.8%	H	75.3%	0.373	41.5
8	f	7, 8, 13, 18, 21, 23, 27, 28	83.0%	H	75.5%	0.195	754.0
9	f	7, 8, 13, 18, 21, 22, 23, 27, 28	84.2%	H	75.1%	0.179	901.7
10	f	7, 8, 10, 13, 18, 21, 22, 23, 27, 28	85.8%	H	75.3%	0.156	866.4

**Table 6:** Results summary for Experiments 1, 2, and 12 and for all databases.

Data set	Experiment	Condition	Run	NF	Acc	Features	TM	TF	*γ*	*C*/*μ*
Diane05	1	Best	e	11	81.94%	7, 8, 10, 12, 14, 18, 21, 22, 24, 27, 30	H	70.00%	0.10	10.00
		NF ≤ 10	d	10	81.67%	3, 8, 10, 14, 15, 19, 21, 22, 24, 30	H	70.00%	0.10	10.00
		NF ≤ 5	e	5	80.00%	7, 8, 18, 21, 30	H	70.00%	0.10	10.00
	2	Best	f	10	85.81%	7, 8, 10, 13, 18, 21, 22, 23, 27, 28	H	75.32%	0.16	866.44
		NF ≤ 10	f	10	85.81%	7, 8, 10, 13, 18, 21, 22, 23, 27, 28	H	75.32%	0.16	866.44
		NF ≤ 5	f	5	81.17%	**7, 13, 21, 23, 29**	H	74.38%	0.01	844.37
	12	Best	b	11	85.57%	7, 10, 12, 13, 15, 16, 18, 22, 23, 24, 27	H	75.16%	0.77	0.46
		NF ≤ 10	e	9	84.56%	5, 6, 10, 12, 13, 19, 21, 22, 29	H	75.21%	0.59	0.47
		NF ≤ 5	e	5	80.21%	1, 10, 12, 13, 29	H	75.94%	1.90	0.49

Diane06	1	Best	e	12	94.17%	1, 8, 9, 10, 11, 12, 14, 18, 20, 21, 24, 26	H	70.00%	0.10	10.00
		NF ≤ 10	e	10	93.06%	1, 8, 9, 10, 11, 14, 20, 21, 24, 26	H	70.00%	0.10	10.00
		NF ≤ 5	e	5	91.94%	1, 9, 11, 14, 24	H	70.00%	0.10	10.00
	2	Best	b	11	95.99%	1, 10, 11, 12, 15, 19, 21, 24, 25, 27, 29	H	73.04%	0.13	697.55
		NF ≤ 10	b	10	95.73%	1, 10, 11, 12, 15, 19, 21, 24, 25, 27	H	72.63%	0.21	705.63
		NF ≤ 5	a	5	92.38%	11, 14, 19, 24, 28	H	74.86%	0.17	281.47
	12	Best	e	17	95.95%	1, 2, 8, 10, 11, 12, 13, 14, 19, 20, 21, 22, 23, 24, 25, 29, 30	H	75.29%	2.45	0.18
		NF ≤ 10	a	9	95.43%	1, 10, 11, 12, 14, 15, 19, 21, 24	H	72.70%	3.45	0.27
		NF ≤ 5	a	5	93.92%	**10, 11, 14, 19, 28**	H	75.33%	2.08	0.34

German	1	Best	d	14	80.00%	1, 2, 3, 5, 6, 10, 12, 14, 15, 16, 17, 18, 19, 20	H	70.00%	0.10	10.00
		NF ≤ 10	e	10	78.67%	1, 2, 3, 5, 6, 7, 10, 12, 18, 19	H	70.00%	0.10	10.00
		NF ≤ 5	a	5	75.33%	1, 3, 5, 8, 19	H	70.00%	0.10	10.00
	2	Best	h	16	81.25%	1, 2, 3, 5, 6, 738310, 11, 12, 14, 15, 17, 18, 19, 20	H	68.04%	0.01	534.75
		NF ≤ 10	a	10	78.99%	1, 2, 3, 4, 6, 8, 11, 12, 15, 19	H	58.63%	0.05	62.26
		NF ≤ 5	c	5	77.16%	1, 3, 5, 7, 12	H	58.45%	0.17	72.52
	12	Best	h	7	80.29%	1, 2, 3, 5, 8, 11, 14	H	58.40%	0.14	0.45
		NF ≤ 10	h	7	80.29%	1, 2, 3, 5, 8, 11, 14	H	58.40%	0.14	0.45
		NF ≤ 5	b	5	77.43%	**1, 5, 12, 13, 14**	H	77.48%	1.41	0.45

Australian	1	Best	h	7	91.35%	1, 6, 8, 10, 12, 13, 14	H	70.00%	0.10	10.00
		NF ≤ 5	h	5	89.42%	6, 8, 9, 10, 14	H	70.00%	0.10	10.00
	2	Best	g	9	93.00%	2, 4, 5, 6, 8, 10, 11, 13, 14	H	70.94%	0.02	180.23
		NF ≤ 5	e	5	92.16%	**2, 4, 6, 8, 9**	H	70.48%	0.58	321.06
	12	Best	j	10	92.86%	2, 3, 4, 5, 6, 8, 10, 11, 12, 14	H	77.61%	2.06	0.34
		NF ≤ 5	b	5	92.00%	3, 4, 5, 8, 10	H	71.02%	2.65	0.34

**Table 7:** Results summary for Experiments 8, 9, 10, 11, and 14 and for all databases.

Dataset	Experiment	ROC area	Condition	Run	NF	*R*	*e*_I_	Features	TM	TF	*γ*	*C*/*μ*
Diane05	8	0.872	*e*_I_ ≤ 10%	c	12	77.1%	9.4%	8, 10, 12, 13, 15, 16, 18, 22, 23, 24, 25, 27	H	77.6%	0.62	108.73
			NF ≤ 5	i	4	64.6%	6.4%	15, 18, 27, 28	H	75.2%	0.03	336.98
	9	0.859	*e*_I_ ≤ 10%	b	10	72.0%	8.8%	7, 8, 10, 12, 13, 19, 22, 23, 25, 29	H	76.3%	0.26	701.29
			NF ≤ 5	c	5	63.0%	7.2%	13, 14, 18, 27, 28	H	76.0 %	0.05	29.51
	10	0.876	*e*_I_ ≤ 10%	d	7	72.0%	8.8%	8, 10, 11, 16, 22, 24, 27	H	76.2%	0.41	19.29
			NF ≤ 5	c	5	64.5%	7.3%	**8, 18, 22, 28, 29**	H	78.5%	0.22	107.89
	11	0.877	*e*_I_ ≤ 10%	a	18	77.0%	8.6%	5, 6, 7, 8, 10, 11, 12, 13, 16, 18, 19, 21, 22, 23, 24, 26, 28, 30	H	77.6%	0.35	25.35
			NF ≤ 5	h	4	60.4%	5.8%	3, 10, 14, 28	H	75.7%	0.21	6.48
	14	0.867	*e*_I_ ≤ 10%	f	13	76.0%	10.1%	5, 7, 12, 13, 16, 19, 21, 22, 23, 24, 25, 27, 28	H	76.0%	0.94	0.49
			NF ≤ 5	h	6	50.3%	3.7%	6, 14, 21, 24, 28, 29	H	76.1%	0.01	0.03

Diane06	8	0.981	*e*_I_ ≤ 10%	e	9	95.3%	8.0%	4, 11, 12, 13, 19, 21, 22, 25, 29	H	76.1%	1.03	157.00
			NF ≤ 5	b	3	68.1%	0.0%	1, 15, 28	H	78.1%	6.27	60.84
	9	0.985	*e*_I_ ≤ 10%	b	7	96.8%	8.0%	5, 7, 10, 11, 21, 24, 25	H	75.5%	1.15	371.95
			NF ≤ 5	b	5	92.9%	2.1%	**7, 11, 21, 25, 28**	H	75.4%	1.37	240.07
	10	0.982	*e*_I_ ≤ 10%	i	18	96.5%	8.3%	1, 3, 4, 6, 8, 11, 12, 13, 15, 16, 17, 19, 21, 22, 23, 24, 25, 30	H	77.1%	3.50	18.67
			NF ≤ 5	c	7	94.2%	8.0%	1, 4, 11, 21, 25, 27, 29	H	75.5%	0.32	57.04
	11	0.982	*e*_I_ ≤ 10%	d	18	95.0%	9.4%	1, 3, 5, 8, 10, 11, 12, 13, 15, 16, 17, 19, 21, 22, 26, 27, 28, 30	H	75.6%	2.39	17.30
			NF ≤ 5	e	10	94.2%	7.3%	1, 3, 4, 7, 11, 14, 16, 19, 22, 28	H	75.5%	7.43	880.32
	14	0.981	*e*_I_ ≤ 10%	h	11	96.2%	7.3%	1, 4, 7, 11, 13, 14, 16, 18, 20, 22, 28	H	75.6%	5.77	0.34
			NF ≤ 5	a	8	93.4%	3.6%	1, 11, 14, 19, 22, 24, 25, 30	H	75.8%	6.01	0.49

German	8	0.765	*e*_I_ ≤ 10%	h	11	57.5%	9.4%	1, 2, 3, 5, 8, 11, 12, 14, 16, 19, 20	H	76.8%	0.02	525.81
			NF ≤ 5	h	5	41.5%	4.9%	**1, 3, 5, 8, 14**	H	58.2%	0.08	669.49
	9	0.757	*e*_I_ ≤ 10%	c	7	50.8%	9.4%	1, 2, 3, 5, 7, 12, 13	H	59.6%	0.27	34.09
			NF ≤ 5	h	4	41.8%	6.5%	1, 3, 5, 8	H	75.3%	0.15	136.06
	10	0.749	*e*_I_ ≤ 10%	e	10	52.2%	9.6%	1, 2, 3, 5, 10, 14, 15, 16, 19, 20	H	72.1%	0.03	773.25
			NF ≤ 5	g	5	50.5%	8.2%	1, 2, 3, 5, 12	H	71.2%	0.17	669.11
	14	0.766	*e*_I_ ≤ 10%	c	4	32.6%	7.5%	1, 2, 5, 7	H	59.1%	0.50	0.47
			NF ≤ 5	c	4	32.6%	7.5%	1, 2, 5, 7	H	59.1%	0.50	0.47

Australian	8	0.964	*e*_I_ ≤ 10%	b	6	97.1%	9.6%	3, 4, 5, 8, 12, 14	H	76.7%	0.53	473.81
			NF ≤ 5	d	5	82.7%	3.5%	4, 8, 9, 13	H	71.9%	9.48	75.61
	9	0.957	*e*_I_ ≤ 10%	g	6	95.3%	9.6%	2, 3, 4, 5, 8, 10	H	71.0%	1.12	8.05
			NF ≤ 5	a	5	90.9%	8.0%	4, 5, 8, 11, 13	H	77.6%	8.50	106.32
	10	0.960	*e*_I_ ≤ 10%	j	5	92.9%	8.8%	6, 8, 9, 13, 14	H	71.5%	1.13	839.90
			NF ≤ 5	j	5	92.9%	8.8%	6, 8, 9, 13, 14	H	71.5%	1.13	839.90
	14	0.967	*e*_I_ ≤ 10%	e	5	93.2%	9.5%	**5, 6, 8, 9, 12 **	H	70.5%	1.30	0.29
			NF ≤ 5	e	5	93.2%	9.5%	**5, 6, 8, 9, 12**	H	70.5%	1.30	0.29
